# Local Juvenile Hormone activity regulates gut homeostasis and tumor growth in adult *Drosophila*

**DOI:** 10.1038/s41598-017-11199-9

**Published:** 2017-09-15

**Authors:** M. M. Rahman, X. Franch-Marro, J. L. Maestro, D. Martin, A. Casali

**Affiliations:** 1 Institute for Research in Biomedicine (IRB Barcelona), The Barcelona Institute of Science and Technology, Baldiri Reixac, 10, 08028 Barcelona, Spain; 2 Institut de Biologia Evolutiva (CSIC-UPF), Barcelona, Spain; 3 Present Address: Department of Molecular Cell Biology, Centre for Cancer Biomedicine, Institute for Cancer Research. Oslo University Hospital, Montebello, N-0379 Oslo Norway

## Abstract

Hormones play essential roles during development and maintaining homeostasis in adult organisms, regulating a plethora of biological processes. Generally, hormones are secreted by glands and perform a systemic action. Here we show that Juvenile Hormones (JHs), insect sesquiterpenoids synthesized by the *corpora allata*, are also synthesized by the adult *Drosophila* gut. This local, gut specific JH activity, is synthesized by and acts on the intestinal stem cell and enteroblast populations, regulating their survival and cellular growth through the JH receptors Gce/Met and the coactivator Tai. Furthermore, we show that this local JH activity is important for damage response and is necessary for intestinal tumor growth driven by activating mutations in Wnt and EGFR/Ras pathways. Together, our results identify JHs as key hormonal regulators of gut homeostasis and open the possibility that analogous hormones may play a similar role in maintaining vertebrate adult intestinal stem cell population and sustaining tumor growth.

## Introduction

Juvenile Hormones (JHs) are versatile hormones, playing major roles during larval development and in adult insects^1, 2^. The most characterized role of JHs is to maintain the larval status between molts after periodic pulses of 20-hydroxyecdysone^3^. In adults, JH modulates many biological processes, such as ovarian maturation, behavior, caste determination, diapause, stress response and life span, among others^1, 2, 4^. Moreover, it has been recently shown that an increase in systemic JHs occurs in females after mating, which induces gut remodeling and proliferation of the intestinal stem cell (ISC) population^5^. These pleiotropic effects of JHs are regulated through multiple pathways.

The adult gut of *Drosophila* is maintained by a population of ISCs. ISCs divide asymmetrically giving rise to another ISC and either an endocrine cell (EE) or an enteroblast (EBs), which in turn differentiates into an enterocyte (ECs)^6-9^. Several signaling pathways such as Notch, Wnt, Jak/STAT, JNK, Nrf2 or Hippo, among others, regulate ISCs self-renewal and proliferation both in normal conditions and in response to damage or injury, ensuring intestinal homeostasis^10–14^. Moreover, misregulation of some of these pathways leads to tumor growth. For example, stem cell-derived clones mutant for the Wg negative regulators Apc and Apc2 and overexpressing the oncogenic form of Ras, Ras^V12^ (hereafter referred as Apc-Ras clones) form overgrowths that show many tumor characteristics^15, 16^.

JHs are acyclic sesquiterpenoids known to be synthesized by the *corpus allatum* (CA), a pair of endocrine glands integrated in the ring gland of insects, which in *Drosophila* is located above the brain hemispheres with its dorsal portion tilted anteriorly^3, 17^. Several genes codify for enzymes required for the biosynthesis of JHs, including *Juvenile hormone acid o-methyltransferase* (*jhamt*). *jhamt* encodes for an enzyme which generates active JHs by transferring a methyl group from S-adenosyl-L-methionine to the carboxyl group of JHs acids at the latest steps of JH biosynthesis^18, 19^. The titer of JH is precisely regulated by many physiological and biochemical processes. Its synthesis is controlled by the nervous system through the secretion of stimulatory and inhibitory neuropeptides (allatotropins and allatostatins respectively)^20^. JHs interact with two bHLH-PAS proteins, Methoprene-tolerant (Met) and its paralog germ cell-expressed (Gce), which act as its receptors^21^. JH binding induces the recruitment of the transcription factor Taiman (Tai) to DNA^22^, triggering the expression of JH-responsive genes such as *Krüppel homolog* 1 (*Kr-H1*)^3^.

Here we describe the adult gut populations of ISCs and EBs as a new source of JHs in *Drosophila*. This local, gut-specific JH production initiates an autocrine loop required for cellular growth and survival of the progenitor cell population in a Met/Gce and Tai-dependent manner. In addition, we show that Apc-Ras-induced tumors fail to grow and survive in the anterior midgut of the adult fly when the gut-specific JH activity is compromised. These results open the interesting possibility that vertebrate adult intestinal stem cells may also be regulated by local hormonal cues, regulating both normal tissue homeostasis and tumor growth.

## Results

### JHAMT expression is required for the survival of aging progenitor cells

**Figure 1:**
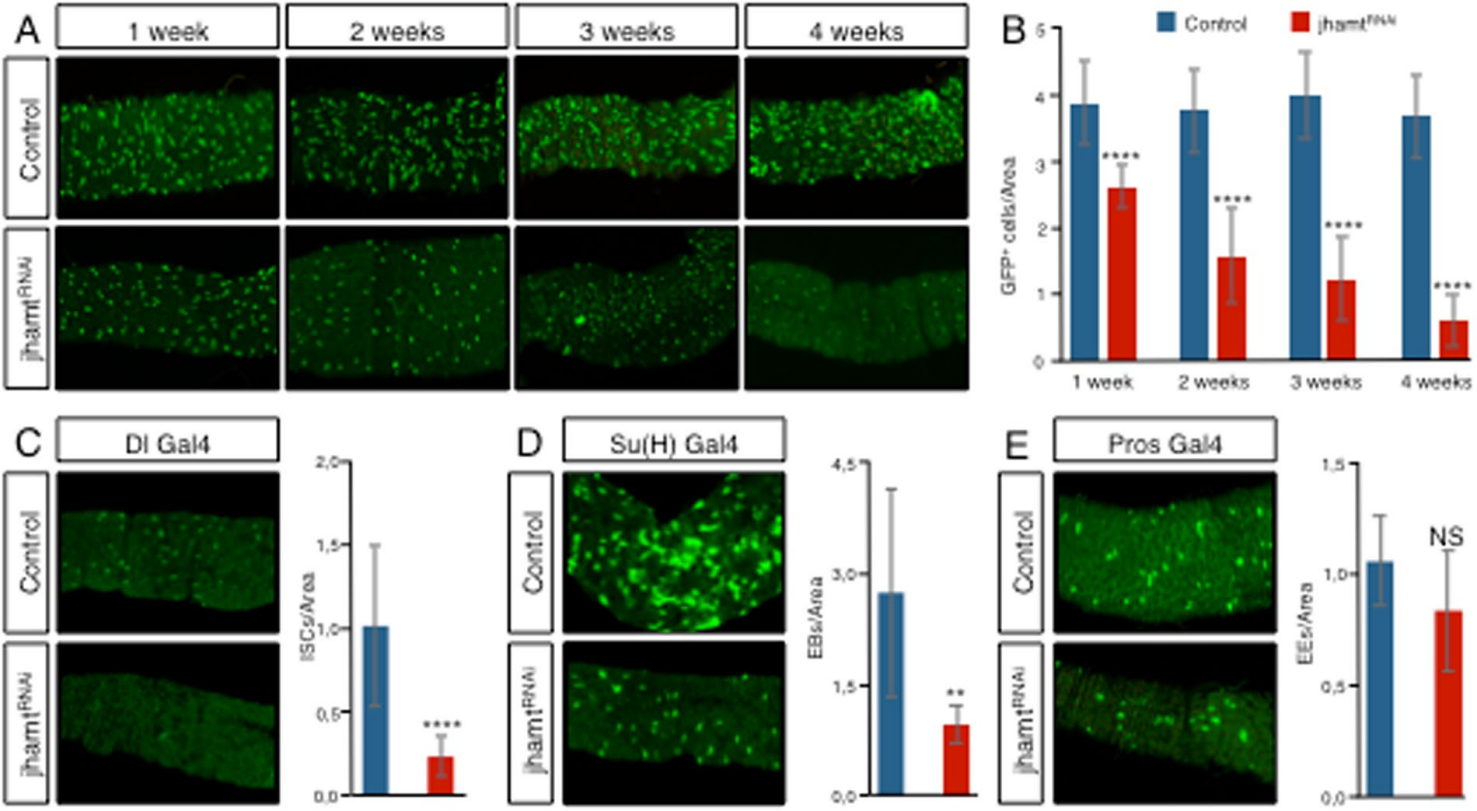
Jhamt activity is required for the maintenance of intestinal homeostasis during aging. (**A**) *jhamt* ^RNAi^ (line 103237) expression in progenitor cells by *esg-Gal4* (marked by *UAS-GFP* expression in green) shows a steady reduction in their number during a four week period at permissive temperature. Images show the same region of the anterior midgut in each condition. Similar results were obtained with the *jhamt* ^RNAi^ line 19172 (Suppl. Fig. 1B). (**B**) Histogram of the mean number of progenitor cells per total gut area. (**C–E**) *jhamt* ^RNAi^ expression on ISCs by *Dl-Gal4* (**C**) and EBs by *Su(H)GBE-Gal4* (**D**) reduce the number of ISCs and EBs respectively, marked by *UAS-GFP* expression (green). In contrast, *jhamt* ^RNAi^ expression in EEs by *pros-Gal4* does not reduce their number (**E**). Histograms show the mean number of ISCs (**C**), EBs (**D**) and EEs (**E**) after two weeks at permissive temperature. Statistical analysis by Wilcoxon Ranked Sum Test: ****p < 0.0001; ***p < 0.001; **p < 0.01; NS, not significant. Error bars show standard deviation. At least 8 guts were analyzed in each condition.

In order to identify new regulators of adult ISC homeostasis, we performed an *in vivo* RNAi screen based on previously selected candidate genes (unpublished work). We expressed each RNAi line upon the *Escargot-Gal4* (*esg-Gal4*) driver, which is specifically expressed in the ISC and EB populations of the adult midgut^6, 7^, controlling the temporal expression by a temperature sensitive allele of Gal80 (Suppl. Fig. 1A). Upon four weeks at permissive temperature, *jhamt* ^RNAi^ expression was able to induce a steady reduction in the number of progenitor cells, in contrast to control flies, which remained constant (Fig. 1A,B and Suppl. Fig. 1B). Jhamt is an O-methyl transferase that converts inactive precursors of JH to active JHs at the very last steps of JH biosynthesis in *Drosophila*.

*jhamt* ^RNAi^ expression under the DeltaGal4 (Dl-Gal4) driver line, which is expressed in the ISCs^23^ was able to significantly reduce the number of ISCs (Fig. 1C and Suppl. Fig. 1A). Similarly, *jhamt* ^RNAi^ expression under the EB-specific Su(H)GBE-Gal4 driver line^23^ was able to reduce the number of EBs (Fig. 1D and Suppl. Fig. 1A). On the contrary, *jhamt* ^RNAi^ expression under the pros-Gal4 driver line, expressed in differentiated EEs, did not significantly affect their numbers (Fig. 1E and Suppl. Fig. 1A). Together, this data uncovers a requirement of jhamt expression for the survival of ISCs and EBs in the aging adult gut.

### Adult gut progenitor cells produce and respond to the JH activity

**Figure 2:**
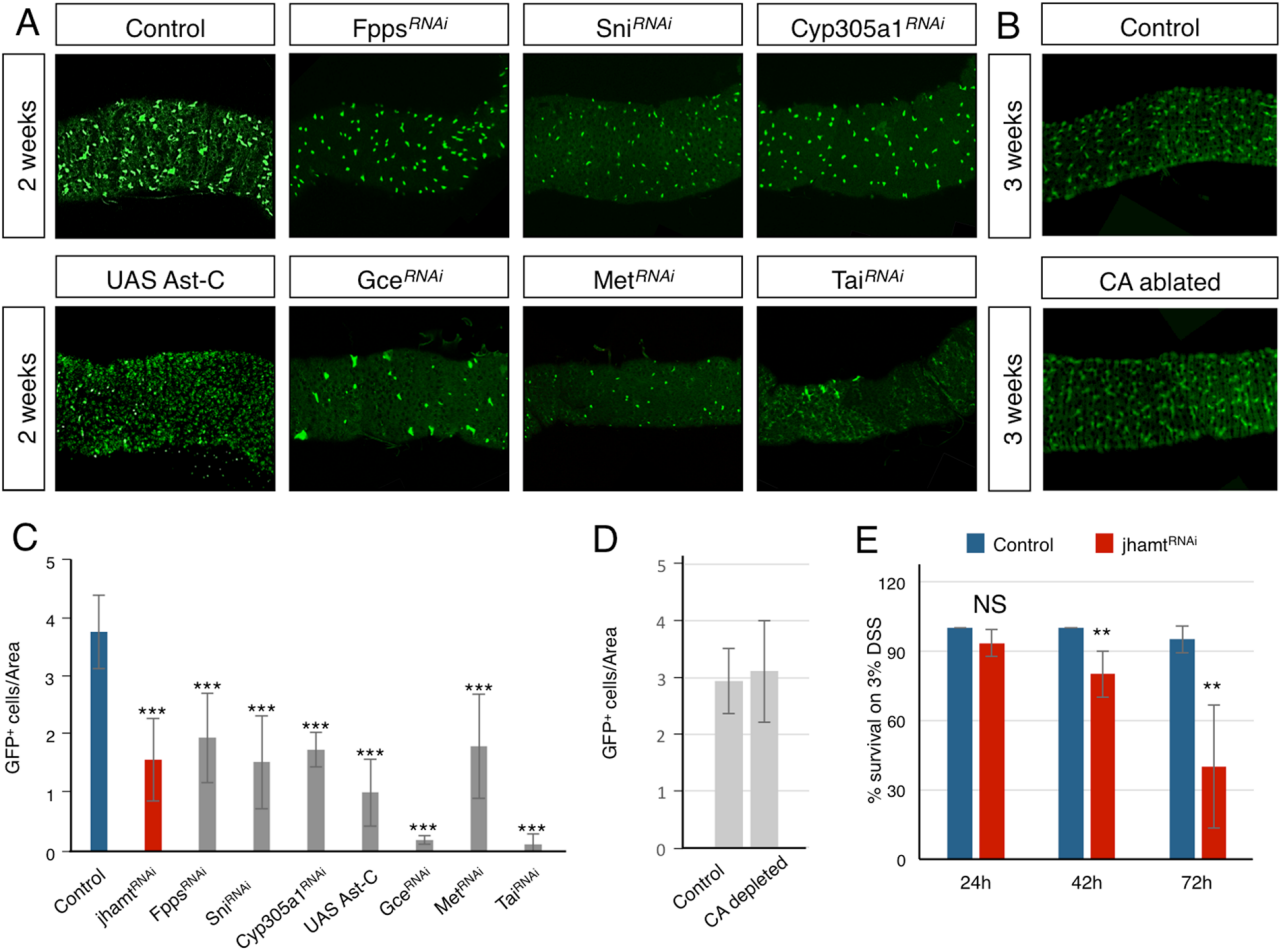
JH is synthesized in the adult gut, which responds to it in an autocrine loop. (**A**) expression in progenitor cells by *esg-Gal4* (marked by UAS-GFP expression in green) of RNAi transgenes against the JH biosynthetic enzymes *Fpps* (line 104362), *sni* (line 106219) and *Cyp305a1* (line 101644), over-expression of *Ast-C*, a known inhibitor of JH biosynthesis, or RNAi mediated knock down of the JH nuclear receptors *gce* (lines 61852 and 26323 together) and *Met* (line 61935), as well as transcriptional coactivator *tai* (line 36095), all lead to reduced number of progenitor cells after two weeks at permissive temperature. Images show the same region of the anterior midgut in each condition. (**B**) Ablation of the CA does not reduce the number of progenitor cells, marked by the expression of *10xSTAT92E-GFP* (in green). (**C**,**D**) Histogram of the mean number of progenitor cells per total gut area. (**E**) Two weeks old flies expressing *jhamt* ^RNAi^ in progenitor cells survive poorly while fed with 3% DSS compared to control flies. 40 control and 30 *jhamt* ^RNAi^ expressing flies were analyzed. Statistical analysis by Wilcoxon Ranked Sum Test: ***p < 0.001; **p < 0.01; NS, not significant. Error bars show standard deviation. At least 5 guts were analyzed in each condition.

In insects, JHs are primarily synthesized and secreted by the CA to perform a systemic action^3^. However, the phenotype observed upon *jhamt* ^RNAi^ expression in ISCs and EBs suggested that the adult gut could act as local source of JH biosynthesis or conversion. Two lines of evidence support this notion. First, we observed that expression of RNAi lines against three other key enzymes required for JH biosynthesis, Farnesyl pyrophosphate synthase (FPPS), the farnesol dehydrogenase Sniffer (Sni) and the JH epoxidase Cyp305a1^24, 25^, under the control of the Esg-Gal4 driver were also able to greatly reduce the number of progenitor cells (Fig. 2A,C and Suppl. Fig. 1B). Second, over-expression of Allatostatin C (Ast-C), a neuropeptide that inhibits JH biosynthesis^26, 27^ and is expressed in the nervous system and in EE cells along the gut^28, 29^ also reduced the number of intestinal progenitor cells (Fig. 2A,C).

Remarkably, this local gut-specific JH activity seemed to have an autocrine effect on the progenitor cell population, as the expression of the RNAi lines against the JH receptors *Met*, its paralog *gce*, and the steroid receptor coactivator *Tai* strongly reduced the number of progenitor cells (Fig. 2A,C and Suppl. Fig. 1B). Noticeably, however, the reduction in the number of progenitor cells was significantly stronger in flies expressing *gce* ^RNAi^ or *tai* ^RNAi^ than in flies expressing *jhamt* ^RNAi^ (Fig. 2A,C), indicating that the effect of JH activity on the survival of the progenitor cell population could depend on both local and systemic JH sources. In order to confirm that the local gut source of JH was able to maintain ISCs and EBs, we eliminated the effect of systemic JH by ablating the CA. Interestingly, the number of ISCs and EBs did not change significantly upon CA ablation (Fig. 2B,D). Together, these results show that the adult *Drosophila* gut is both the source and the target of a local JH activity, which is required to maintain the viability of the aging progenitor cells.

### Physiological relevance of local JH in damage response

We next investigated whether the reduction in the number of progenitor cells produced by the reduction in the local JH synthesis had consequences in coping with oxidative insults, as a measurement of cellular fitness. Flies were fed a 5% sucrose diet supplemented with dextran sulfate sodium (DSS) in order to injure the gut epithelium. As expected, flies expressing *jhamt* ^RNAi^ under the *esg-Gal4* control in progenitor cells showed a reduced life span compared to control flies (Fig. 2E), showing that the reduction in the number of progenitor cells affected gut homeostasis and its ability to cope with stress-induced damage.

### Local JH regulates cell size

**Figure 3:**
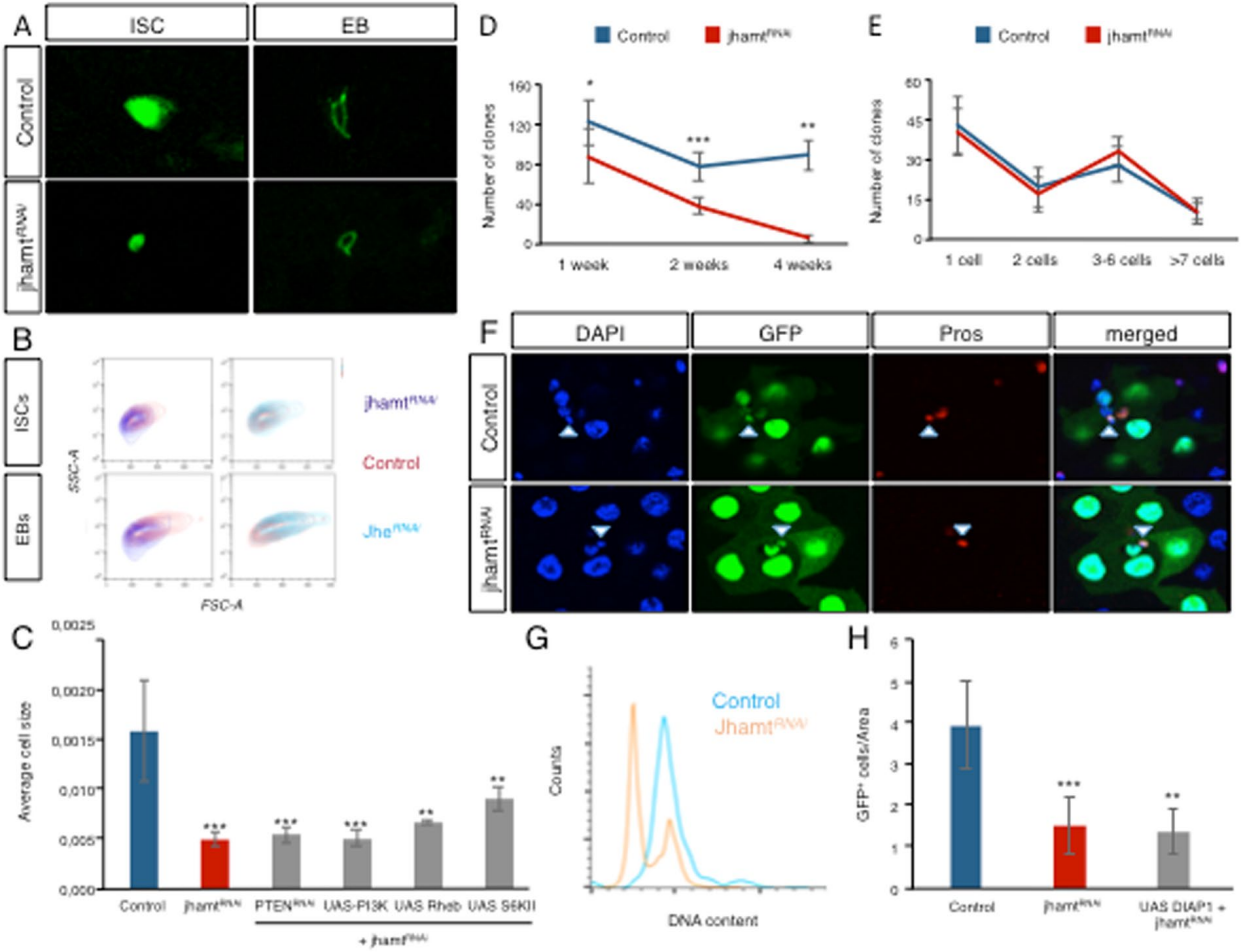
Local JH regulates cell size and cell death. (**A**) Confocal images taken at 63x showing that expression of *jhamt* ^RNAi^ in ISCs (by *Dl-Gal4*) or EBs (by *Su(H)GBE-Gal4*) reduces cell size of ISCs and EBs respectively, compared to control flies. Cells are marked in green by *UAS-GFP* (in ISCs) and *UAS-CD8-GFP* (in EBs). (**B**) Contour plot from sorted cells show that ISCs and EBs expressing *jhamt* ^RNAi^ (in dark blue) are on average smaller than control cells (in red). On contrary, cells expressing *jhe* ^RNAi^ cells (light blue) are slightly bigger. Forward scatter area (FSC-A) on the x-axis and side scatter area (SSC-A) on the y-axis together outline the cell shapes. (**C**) Histogram of the average cell size shows that normal cell size is not restored by IIS/TOR pathway activation in cells expressing *jhamt* ^RNAi^. (**D**,**E**) *Tubulin* > *Gal4* driven MARCM clones expressing *jhamt* ^RNAi^ progressively disappear from the midgut compared to control clones (**D**) but the *jhamt* ^RNAi^-expressing clones that survive show the same size distribution as control clones (**E**). (**F**) control and *jhamt* ^RNAi^ expressing clones (marked by *UAS-GFP* in green) are able to differentiate, showing ECs (marked by big polyploid nuclei stained by DAPI in blue) and EEs (marked by Prospero staining in red). (**G**) DNA content analysis of sorted *jhamt* ^RNAi^ expressing progenitor cells shows a G0 sub phase characteristic of apoptotic cells. (**H**) Histogram of the number of GFP^+^ cells/area shows that co-expression of the antiapoptotic *UAS-Diap1* does not prevent cell death induced by *jhamt* ^RNAi^ expression. Statistical analysis by Wilcoxon Ranked Sum Test: ***p < 0.001; **p < 0.01; NS, not significant. Error bars show standard deviation. At least 5 guts were analyzed in each condition.

We noticed that upon *jhamt* ^RNAi^ expression under the *esg-gal4* driver, progenitor cells became smaller (Fig. 3A). To better characterize the effect of the local JH activity on cellular size, we took advantage of the EB specific marker Su(H)-mCherry^15^. We sorted the ISC (GFP^+^, mCherry^−^) and EB (GFP^+^, mCherry^+^) cell populations and confirmed that upon *jhamt* ^RNAi^ expression cells were consistently smaller on average (Fig. 3B). On the contrary, expression of an RNAi line against *JH esterase* (*JHE*), an enzyme required for the degradation of JH^30^, slightly increased the cellular size of ISCs and EBs (Fig. 3B), implying that local JH is directly involved in cell size regulation. Next we asked whether JH function on cell growth was dependent on the insulin/target or rapamycin (IIS/TOR) pathway, which links nutrient sensing to cellular growth^31, 32^. To activate the pathway we over expressed PI3K, the TOR activator Ras homolog enriched in brain (Rheb) and S6KII, or an RNAi against the negative pathway regulator PTEN, but none of them were able to restore normal progenitor cell size (Fig. 3C) or to increase their survival (Suppl. Fig. 2A) when expressed together with *jhamt* ^RNAi^. We concluded, therefore, that the effect on cell growth mediated by local JH activity is independent or downstream to the IIS/TOR pathway.

In some stem cell populations, as for example most neuroblasts of the central nervous system of *Drosophila*, a reduction in the cellular size precedes a terminal symmetric division during pupal development^33^. We analyzed whether the reduction in the number of progenitor cells upon *jhamt* ^RNAi^ expression was due to the reduction in cell size imposed by lack of JH activity, and a subsequent symmetric division resulting in two EBs. In order to test this possibility, we performed clonal analysis by generating MARCM clones^34^ expressing *jhamt* ^RNAi^ and GFP under the control of the ubiquitous tubulin promoter. We observed a reduction in the number of clones in flies expressing *jhamt* ^RNAi^ compared to control flies (Fig. 3D). However, surviving *jhamt* ^RNAi^ and wild type clones showed similar size distribution at one, two and four weeks after clone induction (Fig. 3E and Suppl. Fig. 2B). These results rule out the possibility that lack of JH activity induces ISCs symmetric division. Remarkably, this data indicates that ISCs surviving *jhamt* ^RNAi^ expression proliferate at the same rate than control ISCs. Moreover, we also observed that clones expressing *jhamt* ^RNAi^ were able to differentiate, as they contained ECs, detected by their large, polyploid nuclei and EEs, detected by Prospero (Pros) staining (Fig. 3F). Therefore, reduction of *jhamt* expression does not seem to impair the potential proliferation or differentiation of the cells that survive.

We next analyzed the DNA content of ISCs and EBs upon *jhamt* ^RNAi^ expression. As previously reported^35^, control flies showed ISCs mostly in G1 phase, with very few events in G2 phase (Fig. 3G). Interestingly, *jhamt* ^RNAi^ expression induced a sub-G0 phase in most ISCs, indicating DNA fragmentation and consequent loss of DNA. This observation suggests that in the absence of JH activity, ISCs may undergo an active process of cell death, consistent with the number reduction previously observed. Overexpression of *Drosophila inhibitor of apoptosis 1* (Diap-1) in *jhamt* ^RNAi^ cells, however, did not restore the wild type number of progenitor cells (Fig. 3H), suggesting that cell death induced by lack of JH must be caspase-independent.

### JH activity is required for tumor growth in the adult anterior midgut

**Figure 4:**
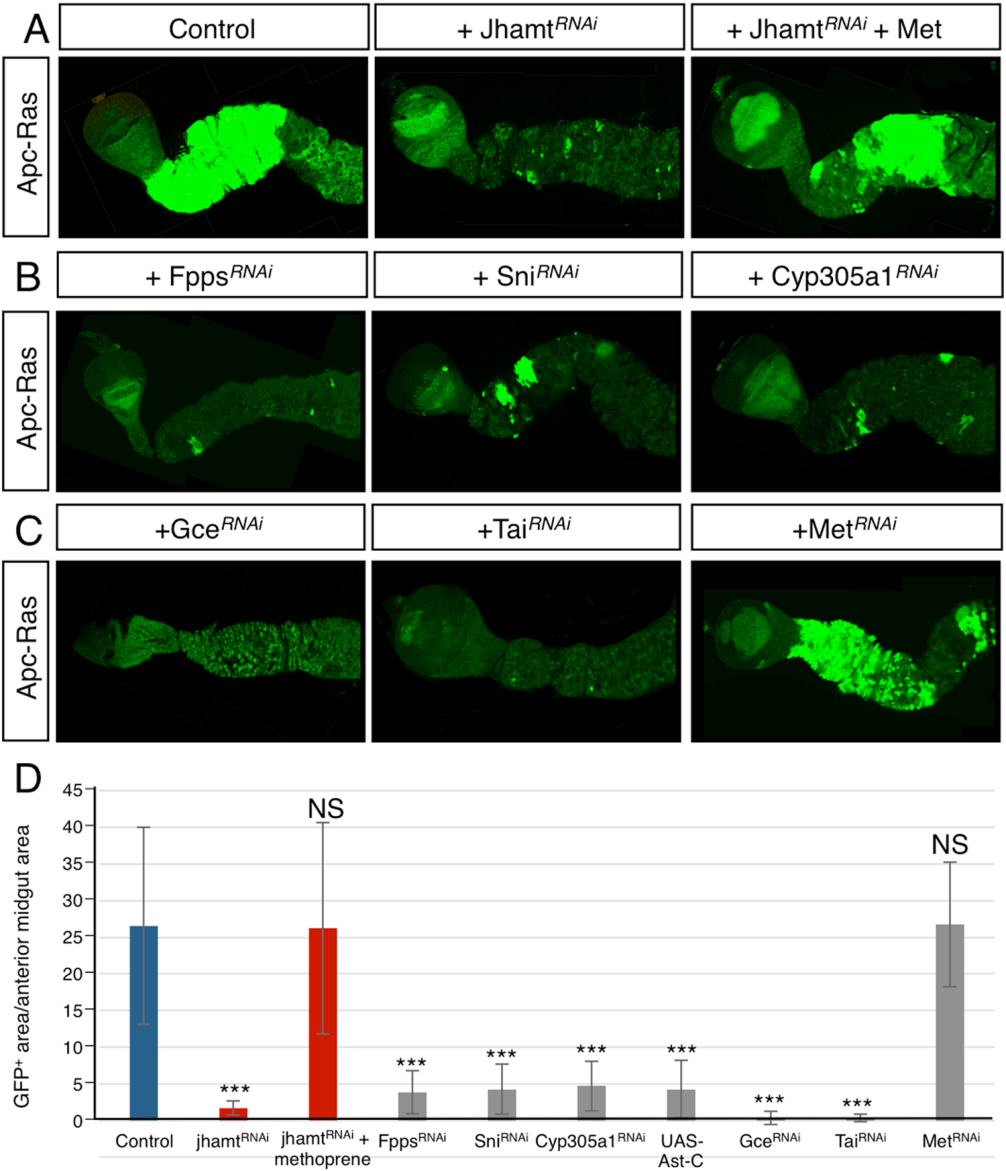
JH activity is required for Apc-Ras induced tumor growth. (**A**) Co-expression of *jhamt* ^RNAi^ in Apc-Ras clones reduces its size after four weeks of clone induction. Treatment with the JH analog methoprene (Met) rescues the growth of Apc-Ras clones. (**B**) RNAi expression of genes of the JH biosynthetic pathway (*Fpps*, *sni* and *Cyp305a1*) reduce the growth of Apc-Ras clones. (**C**) JH receptor *gce* ^RNAi^ expression, as well as *tai* ^RNAI^ expression block the growth of Apc-Ras clones. In contrast, RNAi against the JH receptor Met does not block Apc-Ras clone growth. (**D**) Histogram showing the GFP^+^ area/anterior midgut of the genotypes shown in a-c. Statistical analysis by Wilcoxon Ranked Sum Test: ***p < 0.001; **p < 0.01; NS, not significant. Error bars show standard deviation. At least 6 guts were analyzed in each condition.

We have previously described that mitotic clones mutant for Apc and Apc2 that co-express the oncogenic form of Ras, *UAS-ras* ^*V12*^ under the control of *esg-Gal4* driver line (from now on denoted as Apc-Ras clones), develop as tumor overgrowths in the *Drosophila* intestine. These tumor clones recapitulate several characteristics of human colorectal cancer, serving as a model that has already allowed the identification of previously unknown regulators of intestinal tumorigenesis (Fig. 4A)^15, 36^.

Expression of *jhamt* ^RNAi^ in Apc-Ras clones not only dramatically reduced clone size at four weeks after clone induction (Fig. 4A,D) but also restored life span, which is reduced in flies bearing Apc-Ras clones alone (Suppl. Fig. 3A)^15^. Consistently, administration of the JH analog methoprene restored clone growth (Fig. 4A,D), confirming the role of JH activity in tumor growth. Accordingly, expression of RNAi against the JH biosynthetic enzymes *Fpps*, *sni* and *Cyp305a1* or over-expression of Ast-C also reduced the size of Apc-Ras clones (Fig. 4B,D and Suppl. Fig. 3B).

Downregulation of the JH receptor *gce* or the coactivator *tai* also reduced the growth of Apc-Ras clones (Fig. 4C,D), showing that they directly respond to JH activity. Interestingly, expression of the RNAi line against *Met* was not able to impair tumor growth (Fig. 4C,D), suggesting that in a tumor context Met may not be involved in transducing the JH signal or that its function may be performed by its paralog gce. Taken together, these results suggest that, in *Drosophila*, gut JH activity is required to sustain tumor growth in an autocrine fashion.

## Discussion

JHs regulate many processes during larval development and adult insects. In the adult JHs play many roles, mostly related to reproduction, such as oogenesis, adult male courtship, female sex pheromone production or female gut remodeling in preparation for an increased energy expenditure in egg formation after mating^1, 2, 5, 37, 38^. It is widely accepted that JH is synthesized by the CA, from where it is secreted and performs a systemic action. In this work we identify a previously undescribed gut-specific source of JH biosynthesis, which acts in an autocrine fashion to maintain the survival of progenitor cells in the adult midgut of *Drosophila* during aging. To our knowledge this would be the first time JH is reported to be produced in an adult *Drosophila* organ other than the CA. In the mosquito *Aedes aegypti*, ovaries and male accessory glands synthesize JH^39, 40^, and it has been recently shown that male accessory glands transfer JH to females at mating^41^. Our claim that the adult gut produces an active JH is supported by several lines of evidence. First, RNAi mediated knock down of four genes that codify the enzymes required for JH biosynthesis specifically in midgut progenitor cells show a reduction in the survival of progenitor cells. Second, the same phenotype is observed upon over-expression of the JH biosynthesis inhibitor Ast-C. Third, ablation of adult CA, thus eliminating systemic JH does not affect the number of gut progenitor cells. Finally, growth of Apc-Ras clones expressing *jhamt* ^RNAi^ is rescued by treatment with the JH analog methoprene. However, further work is required to identify specifically which JH activity is being produced by the adult gut.

We report that this adult, gut-specific JH activity acts as a survival factor. JH works by promoting cell growth and suppressing a cell death program and not by regulating cell proliferation or cell differentiation. Cell growth is usually regulated by the IIS/TOR pathway, the components of which are not only essential for cell and organ growth but are also sufficient to accelerate cell growth rate^31^. In fact, activation of the IIS/TOR signaling pathway bypass the cellular effects of starvation such as the arrest of cell growth and DNA replication^31, 42, 43^. JH has been recently shown to regulate the final body size in an IIS/TOR dependent manner^44^, congruent with the notion that IIS signaling may regulate JH synthesis^45^. Our results show that expression of IIS/TOR components are not enough to restore the growth nor the survival of intestinal progenitor cells in absence of the local production of JH activity. Interestingly, the amount of local JH would directly regulate cell size, as over-expression of the RNAi against JHE is enough to increase the average cell size.

Our results also show that cells surviving the lack of JH activity are able to proliferate and differentiate normally. Why do these cells survive? One possibility is that it is a stochastic event related to a variable expression of RNAi transgenes. Another possibility could be that a subtype of progenitor cells evenly distributed along the gut does not require JH to maintain their fitness. However, the progressive phenotype that we observe seems to indicate that eventually all progenitor cells are sensitive to the lack of local JH activity. Overall, our results support the idea that the conjunction of both local and systemic JH activity modulates gut homeostasis. Local gut JH activity in progenitor cells would maintain a competent state to sustain normal functions, regulating its survival during aging. Loss of local JH activity would reduce the fitness of ISCs and EBs over time, reducing their size and eventually triggering a caspase-independent cell death program. Systemic JH would respond to external outputs such as mating^5^, and modify the capacity of the progenitor cells to proliferate, grow, or differentiate, adapting to external cues. Taking into consideration the role of JH preventing metamorphosis during larval development, a picture could emerge from our results in which JH would maintain the progenitor cells in a “juvenile” state in a mostly post-mitotic organism.

Finally, our results also show that local JH is required for intestinal tumor growth in *Drosophila*. Moreover, methoprene treatment is able to sustain growth in the absence of local JH production. These results open the possibility that external cues that increase the systemic level of JH could eventually incise in the rate of growth of tumor cells. In addition, considering the functional homology between JH and retinoic acid, it needs to be investigated in further detail whether hormones produced locally may play a role sustaining tumor growth in colorectal cancer patients.

## Materials and Methods

### Fly stocks

The lines of *jhamt* ^*RNAi*^ (103237 and 19172), *Fpps* ^*RNAi*^ (104362), *Sni* ^*RNAi*^ (106219 and 27342), *Cyp305a1* ^*RNAi*^ (101644 and 51486), *Met* ^*RNAi*^ (100638 and 10801), *gce* ^*RNAi*^ (11176), *tai* ^*RNAi*^ (15709), *Jhe* ^*RNAi*^ (44049) and *PTEN* ^*RNAi*^ (109278) were obtained from VDRC. *Met* ^*RNAi*^ (61935), *gce* ^*RNAi*^ (combination of 61852 and 26323 at the same time), *tai* ^*RNAi*^ (36095), *UAS-PI3K* (8287), *UAS-Rheb* (9689), *UAS-S6KII* (8714), *10xSTAT92E-GFP* (26197), *UAS-EGFP-NiPp1* (23712) and *Aug21Gal4* (30137) were obtained from Bloomington Stock Center. *UAS Diap-1* was a gift from G. Morata. *UAS-AstC* was generated cloning the EcoRI-KpnI fragment from the cDNA clone RH36507 (DGC gold BDGP) into the pUAST vector. Transgene expression together with UAS-GFP was driven by *esg-Gal4*, *Dl-Gal4*, *Su(H)-Gal4* or *pros-Gal4*. Gal4 activity was regulated by *Tub* > *Gal80* ^*ts*^. Flies were crossed at 17 °C, and two day old progeny was transferred to 29 °C for analysis. MARCM clones were generated by a 1hr heat shock at 37 °C of 2–5 days old females and were marked by the tubulin > Gal4 line driving the expression of *UAS GFP* (normal clones). Apc-Ras clones were generated as described previously^15^.

### Immunohistochemistry and microscopy

Adult female flies were dissected in PBS. All the digestive tract was removed and fixed in PBS and 4% electron microscopy grade paraformaldehyde (Polysciences, USA) for 40 minutes. Samples were rinsed 3 times with PBS, 4% BSA, 0.1% Triton X-100 (PBT-BSA), incubated with the primary antibody overnight at 4 °C and with the secondary antibody for 2 hours at room temperature. Finally, the samples were rinsed 3 times with PBT-BSA and mounted in DAPI-containing media (Vectashield, USA). All the steps were performed without mechanical agitation. Primary antibody mouse α-Pros (1:100) was obtained from the Developmental Studies Hybridoma Bank (DSHB). Secondary antibodies were from Invitrogen (USA). Images were obtained on a Leica SPE or Leica SP5 confocal microscopy and processed in Photoshop CS5 (Adobe, USA).

### Corpus allatum ablation

Abolishment of endogenous adult JH production by ablation of the CA was performed by misexpression of the protein phosphatase inhibitor NiPp1 under the CA-specific driver *Aug21-Gal4* ^46, 47^ in a genetic background that contained the ISC and EB marker *10xSTAT92E-GFP* ^48^.

### FACS analysis

20–30 female fly guts were dissected and collected in cold PBS and kept on ice. Guts were digested with 40 mg/ml of Dispase (Invitrogen) for 5 minutes at 37 °C. The guts were then passed through a hypodermic needle using 1 ml syringe repeatedly until the PBS looks slightly cloudy. Cells were checked under microscope to ascertain they were single celled and without clumps and then washed with cold PBS by spinning @1400 rpm/5 minutes/4 °C and re-suspended with cold PBS to a final volume of 200 µl and transferred into a “flow tube” for further nuclear staining and FACS analysis. Cells were sorted with FACS Aria from BD Biosciences and the results were analyzed with Flow Jo V10.0.8 from Tree Star, Inc. Oregon.

### Stress experiments

Flies of appropriate age and genotype were transferred to empty vials and fed with 5% sucrose with or without 3% DSS. The number of dead flies was scored at different time points after the introduction of the DSS.

## Electronic supplementary material

Supplementary Figures
