# Supplementary material for: Local Juvenile Hormone activity regulates gut homeostasis and tumor growth in adult Drosophila
Source: Sci Rep. 2017 Sep 15;7:11677. doi: 10.1038/s41598-017-11199-9 (PMC5600977; doi:10.1038/s41598-017-11199-9)
Supplement: Supplementary file 1 — Supplementary Figures [file 41598_2017_11199_MOESM1_ESM.pdf]

Supplementary Information

Local Juvenile Hormone activity regulates gut homeostasis and tumor growth in adult *Drosophila*

M. M. Rahman[1,*], X. Franch-Marro[2], J.L. Maestro[2], D. Martin[2] and A. Casali[1,3]

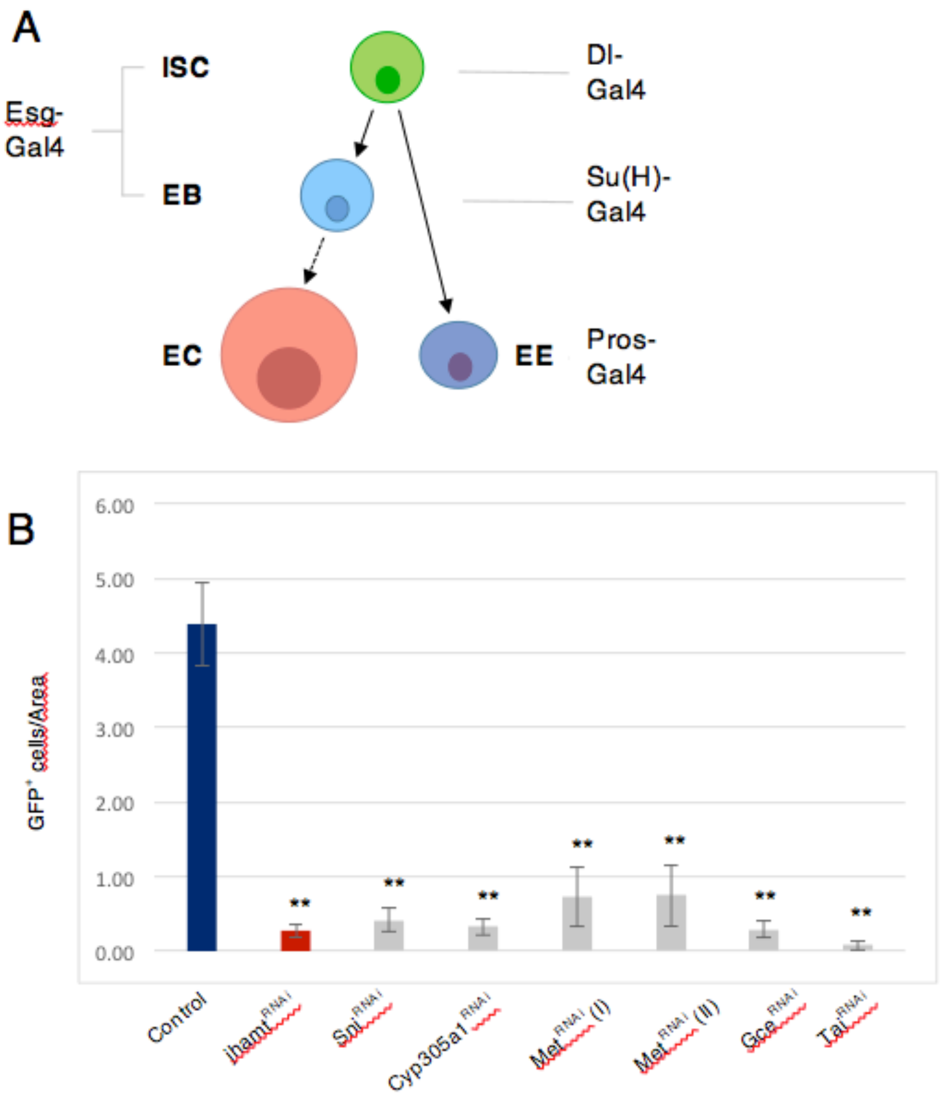

Supplementary Figure 1. **a**. Diagram of normal adult midgut division differentiation process and the Gal4 lines that drive the expression in each cell type used in this study. ISC Intestinal Stem Cell, EB enteroblast, EC enterocyte, EE enteroendocrine cell. **b**, Histogram of the mean number of progenitor cells per total gut area. RNAi transgenes against *Jhamt* (19172)), *sni* (line 27342), *Cyp305a1* (line 51486), *Met* (lines 100638 and 10801), *gce* (line 11176) and *tai* (line 15709) were tested to confirm the reduction in the number of progenitor cells was not an off-target effect. Statistical analysis by Wilcoxon Ranked Sum Test: ** $p < 0.01$. Error bars show standard deviation. At least 4 guts were analyzed in each condition.

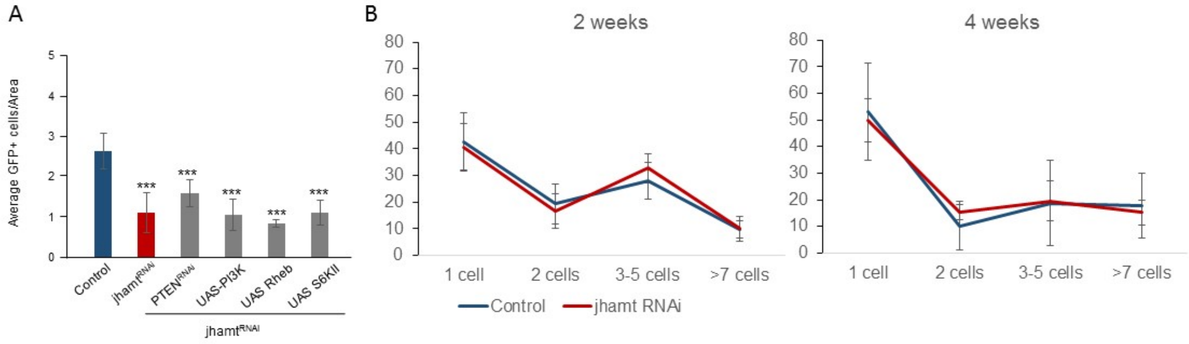

Supplementary Figure 2. **a**, Histogram of the average number of GFP^+ cells per gut area is not restored by IIS/TOR pathway activation in cells expressing *jhamt*^{RNAi}. **b**, *Tubulin>Gal4* driven MARCM clones expressing *jhamt*^{RNAi} that survive show the same size distribution than control clones two and four weeks after clone induction. Statistical analysis by Wilcoxon Ranked Sum Test: *** p<0.001; ** p<0.01. Error bars show standard deviation. At least 6 guts were analyzed in each condition.

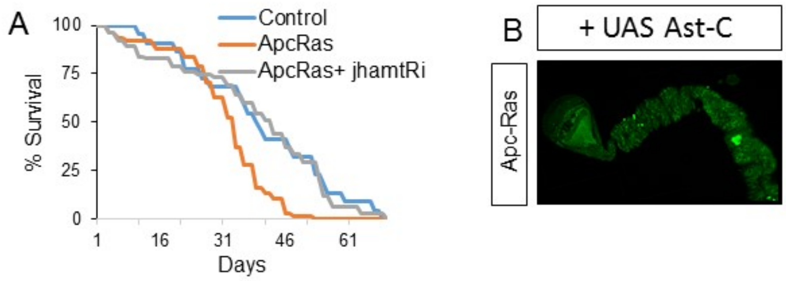

Supplementary Figure 3. Effects of JH in tumor growth. **a**, life span of flies bearing Apc-Ras clones is restored to wild-type levels after co-expression of *jhamt*^{RNAi}. Survival of flies were scored every alternate day while changing the food and maintained at 25ºC until all flies died. **b**, Over-expression the inhibitory neuropeptide Allatostatin-C in Apc-Ras clones suppresses growth (marked by GFP in green).
